# Development of an HIV-1 Microbicide Based on *Caulobacter crescentus*: Blocking Infection by High-Density Display of Virus Entry Inhibitors

**DOI:** 10.1371/journal.pone.0065965

**Published:** 2013-06-19

**Authors:** Christina Farr, John F. Nomellini, Evan Ailon, Iryna Shanina, Sassan Sangsari, Lisa A. Cavacini, John Smit, Marc S. Horwitz

**Affiliations:** 1 Department of Microbiology and Immunology, The University of British Columbia, Vancouver, British Columbia, Canada; 2 Beth Israel Deaconess Medical Center, Boston, Massachusetts, United States of America; Scripps Research Institute, United States of America

## Abstract

The HIV/AIDS pandemic remains an enormous global health concern. Despite effective prevention options, 2.6 million new infections occur annually, with women in developing countries accounting for more than half of these infections. New prevention strategies that can be used by women are urgently needed. Topical microbicides specific for HIV-1 represent a promising prevention strategy. Conceptually, using harmless bacteria to display peptides or proteins capable of blocking entry provides an inexpensive approach to microbicide development. To avoid the potential pitfalls of engineering commensal bacteria, our strategy is to genetically display infection inhibitors on a non-native bacterium and rely on topical application of stabilized bacteria before potential virus exposure. Due to the high density cell-surface display capabilities and the inherent low toxicity of the bacterium, the S-layer mediated protein display capabilities of the non-pathogenic bacterium *Caulobacter crescentus* has been exploited for this approach. We have demonstrated that *C. crescentus* displaying MIP1α or CD4 interfered with the virus entry pathway and provided significant protection from HIV-1 pseudovirus representing clade B in a standard single cycle infection assay. Here we have expanded our *C. crescentus* based microbicide approach with additional and diverse classes of natural and synthetic inhibitors of the HIV-1 entry pathway. All display constructs provided variable but significant protection from HIV-1 infection; some with protection as high as 70%. Further, we describe protection from infection with additional viral clades. These findings indicate the significant potential for engineering *C. crescentus* to be an effective and readily adaptable HIV-1 microbicide platform.

## Introduction

The HIV/AIDS pandemic is one of the largest global health concerns, with over 32 million people worldwide living with an HIV infection [1]. The majority of new infections occur through sexual transmission, with 2.6 million new infections annually [1], [2]. Sexual transmission of HIV can be prevented by the use of condoms, but women in developing countries do not always have the option to insist on condom use, often due to cultural or religious practices [2]. As such, women over the age of 15 in developing countries account for the majority of new HIV-1 infections [1], [2]. The development of prevention strategies that can be used by women is urgently needed. With the difficulties in developing an efficacious vaccine, alternative prevention options are required.

Microbicides are drug products that are topically applied to mucosal surfaces to prevent infection [3]. They are able to fill the need for female controlled prevention, as they can be administered without partner knowledge, and are able to maintain efficacy for extended periods of time. Currently, no microbicides for HIV are on the market, but approximately 50 candidates are under development [3]–[5]. Many of these are non-specific and/or expensive to produce, which may limit the clinical effectiveness and practicality for delivery to third world populations [3], [6]. To counteract these difficulties, some efforts have focussed on engineering bacteria to display HIV blocking agents [6]–[8]. While most of these approaches have used commensal vaginal bacteria, such as *Lactobacillus*, use of these recombinant bacteria would rely on their ability to colonize the vaginal tract and maintain expression of the protein for extended periods of time, which has shown limited success [6]–[8]. Issues with expression and secretion of larger proteins, as well as concerns about eradication of the bacteria should unwanted side effects occur, suggest the use of *Lactobacillus* species in microbicide formulations may be problematic [6]–[8].

In contrast, we have engineered an alternative bacterium-based microbicide strategy that does not require colonization or maintenance of the bacteria within the individual. Using the non-pathogenic, freshwater bacterium *Caulobacter crescentus* [9], [10] we have generated a system to insert foreign protein sequences into the surface (S)-layer protein RsaA of *C. crescentus* [11]–[15]. Native expression levels of RsaA exceeds 30% of total cell protein and the insertion of foreign proteins often has minimal impact on expression and assembly of RsaA into the ordered S-layer structure [16]. Although difficult to assess in every instance, the proteins generally retain their function when displayed within the S-layer [11]–[15]. Proteins of a wide range of sizes can be expressed and displayed within the S-layer [12], [15]. In addition to the protein expression capabilities, *C. crescentus* are unable to grow at temperature above 30°C or at the salt concentrations found in human tissue, indicating these bacteria will not colonize a human host. Further, the lipopolysaccharide of *C. crescentus* has an unusually low endotoxin response, and no obvious adverse effects when injected intraperitoneally into mice [17], [18]. In our view these features make it an ideal candidate for development into a microbicide [17]–[19].

In a previous paper we described work with HIV-1 clade B pseudovirus. Clade B causes approximately 10% of all HIV-1 infections and is most common in the America’s and Western Europe. Here we expanded our work to include HIV-1 clade C pseudovirus, which is the main cause of the HIV-1 pandemic, is responsible for 52% of all infections worldwide, and is distributed in Sub-Saharan Africa and other developing countries. Taken together, these experiments address over 60% of all the HIV-1 infections circulating. HIV-1 clades A and D and A/D recombinant were also used in preliminary studies; these represent the next most common viral clades, and are more locally distributed [20], [21].

The *C. crescentus* protein display system was utilized previously to express MIP1α or domain 1 of CD4 in the S-layer [9]. Expression of either MIP1α or CD4 individually provided significant protection from infection with several variants of HIV-1 pseudovirus representing clade B in a standard single cycle infection assay [9]. Herein, we expand on those findings to show protection from infection with HIV-1 pseudovirus representing clade C. In addition, *C. crescentus* constructs that express the CD4 mimetic CD4M33F23, multiple anti-HIV-1 lectins, as well as three variants of gp41 fusion inhibitors are introduced. These agents represent a range in potential with both high and low expectations for successful display as well as blocking HIV-1 infection. Together, they have allowed us to better define the limitations of agents that can be utilized within *C. crescentus* based microbicides. All the proteins were expressed individually within the S-layer, and each *C. crescentus* construct provided variable, yet significant protection from HIV-1 pseudoviruses representing clades B and C, uncovering the large potential for efficacy and function using this *C. crescentus* based microbicide strategy. The anti-HIV lectins were the most effective at preventing HIV infection, providing up to a 70% decrease in infection levels.

## Materials and Methods

### Bacterial Strains and Growth Conditions

*Escherichia coli* strain DH5α (Invitrogen, Carlsbad, CA) was grown at 37°C in Luria Broth (1% tryptone, 0.5% NaCl, 0.5% yeast extract). *C. crescentus* strains were grown in PYE medium (0.2% peptone, 0.1% yeast extract, 0.01% CaCl_2_, 0.02% MgSO_4_) at 30°C. For growth on solid medium, agar was added to 1.3%. JS 4026 has been described [16] and is a derivative of *C. crescentus* CB2A [22]. *C. crescentus* strain JS4038 was made defective in capsular polysaccharide synthesis [23]. It is a modification of JS4026 in which a GDP-L-fucose synthase gene (comparable to the gene annotated as CCNA00471 in *C. crescentus* NA1000) [24] has been inactivated by introduction of an internal deletion. The oligos JN471-1 (CCGCATCGAGCATATTTATCAAGATCC) and JN471-52 (GTATCGGTGATGATCTCGCCCTTG) were used to amplify by PCR a 1721 bp fragment from *C. crescentus* strain CB2A. This PCR product contained 900 bp upstream of the gene and the first 821 bp of the 967 bp GDP-L-fucose synthase gene. After blunt ligation into EcoRV cut pBSKIIEEH [25] the 215 bp Bstx1– Sma1 segment was removed, blunted and religated to make an internal deletion in the fucose synthase gene. This ∼1500 bp segment was moved into pKmobSacB [26] as an EcoR1/HindIII fragment. Clones were screened for first and second crosses with the oligos JN 471-31 (CTGCTGATCGGTGATCCGACGAAG) and JN 471-52. A clone demonstrating a ∼750 bp PCR product (indicating replacement of the chromosomal gene with the deletion version) and loss of mucoid colony appearance on PYE media supplemented with 3% sucrose [24] was named JS4038.

When needed, media contained chloramphenicol (CM) at 20 µg/ml (*E. coli*) or 2 µg/ml (*C. crescentus*). Electroporation of *C. crescentus* was performed as previously described [27].

### Plasmids and DNA Manipulations

The clade B and clade C HIV-1 panels were obtained from the NIH AIDS Reagent and Reference Program. *E. coli* with plasmids containing the viral DNA cassettes were grown as described [9]. Plasmids were purified using a large scale plasmid isolation procedure [28]. The Nucleic Acid Protein Service Unit of the University of British Columbia provided DNA sequencing. Small-scale plasmid isolations were done using EZ-10 spin mini plasmid kits (Bio Basic Inc). DNA fragments were recovered from agarose gels using a QIAEX II gel extraction kit (QIAGEN).

### Preparation of *C. crescentus* Displaying Chimeric S-layer Proteins

The MIP1α and CD4 gene segments have been described [9].

All other gene segments were synthesized by commercial sources with codon usage adapted for *C. crescentus*. The amino acid sequence specified for each are indicated in Table 1. In addition, the synthesized DNA segments specified BglII and SpeI restriction sites on the 5′ side and an NheI site on the 3′ end. This permitted directional cloning into p4ARsaA(723)/GSCC digested with BglII and NheI [15].

**Table 1:** Displayed Protein Sequences.

Displayed segment	Amino acid sequence of displayed segment	Size (# of amino acids)	Name of caulobacter display construct*	Reference
Mip1α	APLAADTPTACCFSYTSRQIPQNFIADYFETSSQCSKPSVIFLTKRGRQVCADPSEEWVQKYVSDLELSA	70	Cc-MIP1a	[9]
CD4	GDTVELTCTASQKKSIQFHWKNSNQIKILGNQGSFLTKGPSKLNDRADSRRSLWDQGNFPLIIKNLKIEDSDTYICEVEDQ	81	Cc-CD4	[9]
CD4 M33 F23	NLHFCQLRCKSLGLLGKCAGSFCACV	26	Cc-CD4M33F23	[31]
Cyanovirin-N	LGKFSQTCYNSAIQGSVLTSTCERTNGGYNTSSIDLNSVIENVDGSLKWQGSNFIETCRNTQLAGSSELAAECKTRAQQFVSTKINLDDHIANIDGTLKYE	101	Cc-CV	[32]
Microvirin	MPNFSHTCSSINYDPDSTILSAECQARDGEWLPTELRLSDHIGNIDGELQFGDQNFQETCQDCHLEFGDGEQSVWLVCTCQTMDGEWKSTQILLDSQIDNNDSQLEIG	108	Cc-MV	[36]
Griffithsin	SLTHRKFGGSGGSPFSGLSSIAVRSGSYLDAIIIDGVHHGGSGGNLSPTFTFGSGEYISNMTIRSGDYIDNISFETNMGRRFGPYGGSGGSANTLSNVKVIQINGSAGDYLDSLDIYYEQY	121	Cc-Gr	[39]
Fuzeon	MYTSLIHSLIEESQNQQEKNEQELLELDKWASLWNWFM	38	Cc-Fz	[41]
T1249	WQEWEQKITALLEQAQIQQEKNEYELQKLDKWASLWEWF	39	Cc-T1249	[43]
C52	NHTTWMEWDREINNYTSLIHSLIEESQNQQEKNEQELLELDKWASLWNWFNI	52	Cc-C52	[45]

Following ligation, plasmids were introduced into *E. coli* by electroporation. Inserted sequences were confirmed by DNA sequencing before transfer to *C. crescentus* by electroporation.

### Protein Analysis

S-layer proteins were prepared by a low-pH extraction method [29]. Proteins were visualized using sodium dodecyl sulfate-polyacrylamide gel electrophoresis (SDS-PAGE) using 7.5% separating gels and staining with Coomassie Brilliant Blue R.

### Preparation of *C. crescentus* Cells for Binding Assay

*C. crescentus* S-layer display constructs were grown in PYE medium to an optical density at 600 nm of approximately 1 (3.1×10^9^ cells/ml). Cells were centrifuged and suspended in water. This was repeated and cell density was adjusted to 1×10^10^ cells/ml for binding experiments.

### Pseudovirus Transfection

One day prior to transfection 3.6×10^6^ 293T cells were seeded in a 10 cm Corning plate, using complete DMEM (1% penicillin/streptomycin and 7.5% FBS). Transfection was performed using the jetPrime kit (Polyplus Transfection). 12 µg rev/env clone and 24 µg HIV-1-env-deficient backbone were diluted in 1.5 mL buffer. After vortex mixing, 30 µL of jetPrime reagent was added and the mixture was incubated for 10 min at room temperature, added to the 293T cells in FBS- and antibiotic-free medium and incubated for 4–6 h. The medium was then replaced with antibiotic-free DMEM containing 7.5% FBS and the cells were incubated for 48 h at 37°C. The virus produced was harvested and collected using a 0.45 µm syringe filter and stored at −80°C.

### Virus Titration

Serial dilutions of virus were prepared in 96-well plates using medium containing 75 µg/ml DEAE-dextran. TZM-bl cells were adjusted to a concentration of 2×10^5^ cells/ml and added to each well. The plates were maintained at 37°C and 5% CO_2_ for 48 h. Infection of cells was measured indirectly using a Mammalian β-galactosidase assay kit (PIERCE) followed by absorbance reading at 415 nm. An absorbance of greater than 0.2 was considered a positive infection. The Tissue Culture Infectious Dose (TCID50) per ml was determined for each viral stock by identifying the dilution of virus in which 50% of the TZM-bl cells were infected, as measured by the presence of β-galactosidase. It was determined that doses of 200 TCID50 were sufficient for experimentation.

### Virus Blocking Experiments

The *C. crescentus* constructs were grown at 30°C in PYE medium with 2 µg/ml chloramphenicol 1–2 days before the experiment, prepared in sterile water as described above and diluted to 5×10^9^ cells/ml immediately prior to experimental setup. Experiments were carried out in quadruplicate wells of 96-well plates. The volume of virus added was determined by calculating the 200TCID50 value. 1×10^8^ *C. crescentus* cells were added to each well. The virus and *C. crescentus* constructs were incubated for 1 hour at 37°C before adding 10,000 TZM-bl cells and 75 µg/ml DEAE-dextran to each well. Cc-MIP1α was incubated with the TZM-bl cells for 1 hour before pseudovirus was added. After an overnight incubation at 37°C and 5% CO_2_ the plates were centrifuged at 800 rpm for 5 min and medium was changed in all wells. After 24 h the level of infection was determined by β-galactosidase assay kit as described above. Data are presented and determined as a percentage of infection of the untreated control infection wells with the background from uninfected TZM-bl cells subtracted. gp41 monoclonal antibody 2F5 or gp120 monoclonal antibody (IgG1b12) were from the NIH AIDS Reagent and Reference Program.

### Statistical Analysis

Statistical analysis was performed with Prism GraphPad software. Results are reported as mean+SEM. Student’s t test or ANOVA were used to evaluate the significance of differences between groups. Experiments were repeated a minimum of three times.

## Results

### Expression of Recombinant Proteins on the Surface of *C. crescentus*

**Figure 1:**
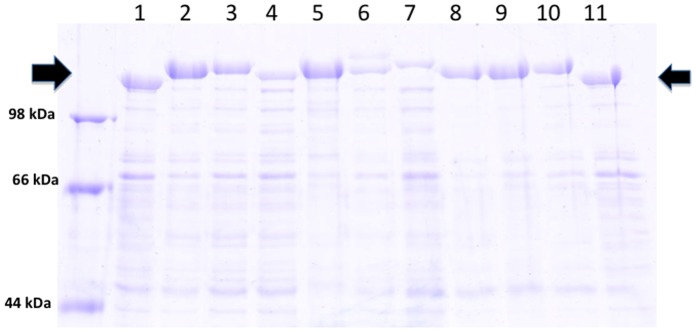
Recombinant S-Layer protein gel. Commassie blue stained 7.5% SDS-PAGE of normalized, low pH extracted protein from *C. crescentus* strain JS 4038 containing RsaA plasmids. Arrows indicate the location of the S-layer protein. 1) Cc-Control (no insert); 2) Cc-MIP1α; 3) Cc-CD4; 4) Cc-CD4M33F23; 5) Cc-CV; 6) Cc-MV; 7) Cc-Gr; 8) Cc-Fz; 9) Cc-T1249; 10) Cc-C52; 11) Cc-Control (no insert).

Several classes of anti-HIV proteins were identified from the literature, including natural proteins, anti-HIV lectins and synthetic fusion inhibitors. These proteins target various parts of the HIV infection process, and are diverse in size and composition. Using an expression vector for *C. crescentus* carrying restriction sites for insertion of genetic material at a site corresponding to amino acid 723 of the S-layer protein [15], recombinant *C. crescentus* that display each of these proteins within the S-layer were generated. The sequence of each expression vector was confirmed prior to transformation into *C. crescentus*. A low pH extraction method (which has been a reliable method to monitor export, assembly and surface attachment of S-layer proteins) and SDS-PAGE was used to measure the expression levels for the chimeric S-layer proteins. Although not quantitated, all constructs resulted in expression of substantial quantities of protein, and demonstrated a size shift by protein gel analysis, indicating that the recombinant protein was expressed. In most cases, the level of recombinant chimeric S-layer protein was comparable to native protein expression (Fig. 1). We previously demonstrated surface expression of recombinant S-layer protein and display of inserted proteins with antibodies directed to CD4 and MIP1α [9]. Herein we generated a number of new constructs, summarized in Table 1.

### Inhibition of Clade C HIV-1 Pseudovirus Infection with Recombinant Caulobacter Expressing MIP1α

**Figure 2:**
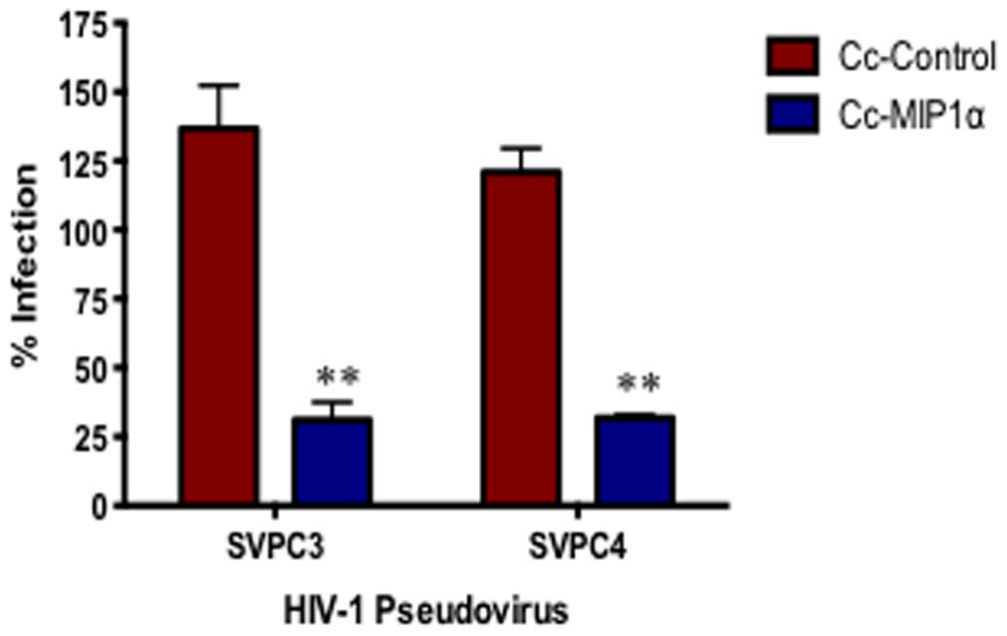
Cc-MIP1α viral blocking assay. Cc-Control and Cc-MIP1α were incubated with TZM-bl cells and 200 TCID50 SVPC3 or SVPC4 pseudovirus for 48 hours. HIV-1 infection was measured indirectly using a mammalian β-galactosidase assay. Infection rate is shown as a percentage and was normalized with virus+TZM-bl cells with the background for uninfected TZM-bl cells subtracted out. The assay was set-up in quadruplicate and repeated a minimum of 3 times. **p<0.001.

MIP1α is the natural ligand for the HIV-1 coreceptor CCR5, which is the most common coreceptor used during initial HIV-1 infection [30]. We have previously generated recombinant Cc-MIP1α and found that it was sufficient to block infection with an HIV-1 pseudovirus representing clade B by 35–78% in single cycle infection assays [9]. To further investigate the relevance of *C. crescentus* display for HIV-1 prevention we measured inhibition of infection with HIV-1 pseudovirus representing clade C. Cc-MIP1α cells (10^8^) were preincubated with TZM-bl cells, then either SVPC3 or SVPC4 pseudovirus was added. Previously, dose response experiments demonstrated that 10^8^ *C. crescentus* was sufficient to block HIV-1 infection with clade B viruses [9]. Due to the small volumes used for these studies, use of higher numbers of *C. crescentus* was not feasible. We found that Cc-MIP1α provided statistically significant protection from infection when compared to Cc-Control with each clade C pseudovirus (Fig. 2). In particular, Cc-MIP1α reduced infection by 52–84% across the two strains. In addition, preliminary experiments with pseudoviruses representing clades A, D, and the use of an A/D recombinant virus also indicated that Cc-MIP1α is able to provide protection from infection with these viruses (data not shown). These findings are similar to what we have previously observed using clade B viruses, and indicate that our previous work with Cc-MIP1α can be extended to include protection from clade C and likely several other viral clades.

### Inhibition of HIV-1 pseudovirus Infection with Recombinant *C. crescentus* Expressing Domain 1 of CD4 or CD4-mimetic with the S-layer

**Figure 3:**
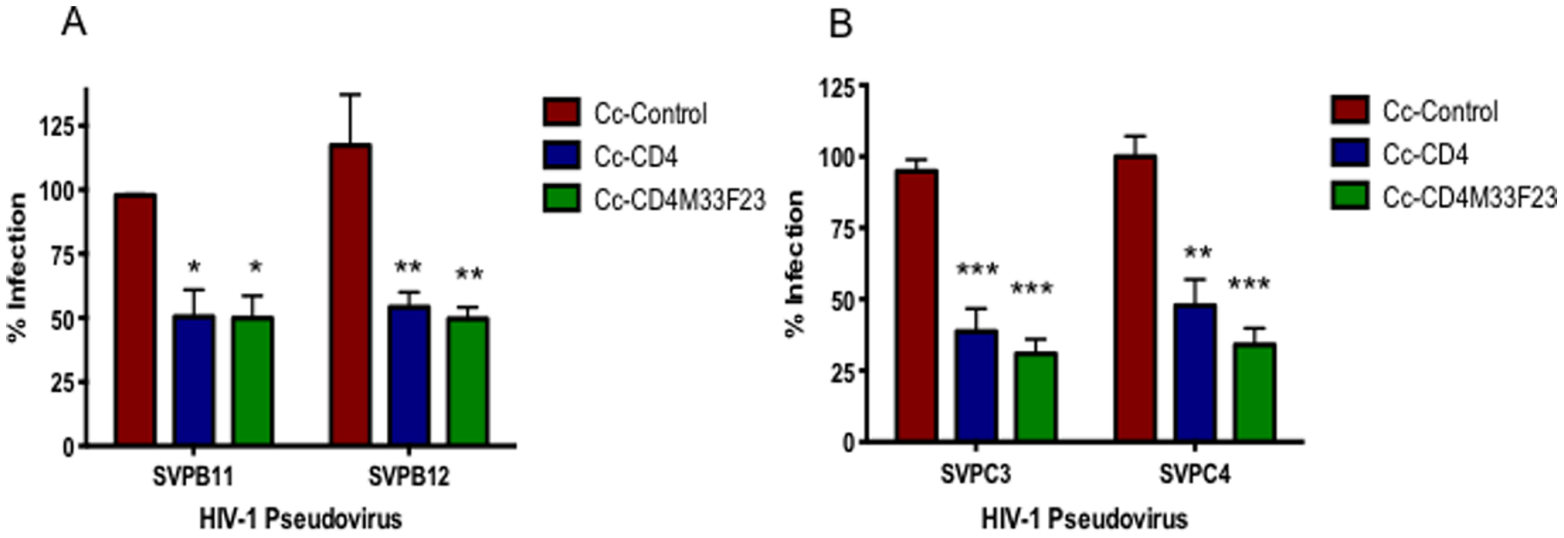
Viral blocking assay with Cc-CD4 and Cc-CD4M33F23. Cc-Control, Cc-CD4 and Cc-CD4M33F23 were incubated for 48 hours with TZM-bl cells and 200TCID50 clade B (SVPB11, SVPB12) or clade C (SVPC3, SVPC4) HIV-1 pseudovirus. HIV-1 infection was measured indirectly using a mammalian β-galactosidase assay. Infection rate is given as a percentage and was normalized to wells containing virus+TZM-bl cells with the background for uninfected TZM-bl cells subtracted out. The assay was set-up in quadruplicate and repeated a minimum of 3 times. *p<0.01, **p<0.001.

Previously, we generated recombinant Cc-CD4 and demonstrated Cc-CD4 was able to provide 22–56% protection from infection with HIV-1 pseudovirus representing clade B [9]. Cc-CD4M33F23 was generated and compared to Cc-CD4 using clade B and C pseudoviruses. CD4M33F23 is a CD4 mimetic that has been shown to be more effective than CD4 and to interact with gp120 and inhibit infection of a wide range of HIV-1 isolates [31]. Co-incubating Cc-CD4 with HIV-1 pseudovirus representing clade C resulted in a significant inhibition of infection of TZM-bl cells (Fig. 3). Infection was inhibited at levels that were similar to those previously observed with the clade B pseudovirus. Cc-CD4M33F23 also provided significant protection from infection with either clade B or clade C viruses (Fig. 3). Similarly, protection levels were maintained with clade D and a clade A/D recombinant virus (data not shown). With most of the pseudoviruses, Cc-CD4M33F23 was more effective than Cc-CD4 in preventing HIV-1 infection, but the difference between these two constructs was not statistically significant.

### Anti-HIV Lectins Provide Significant Protection from HIV-1 Infection

**Figure 4:**
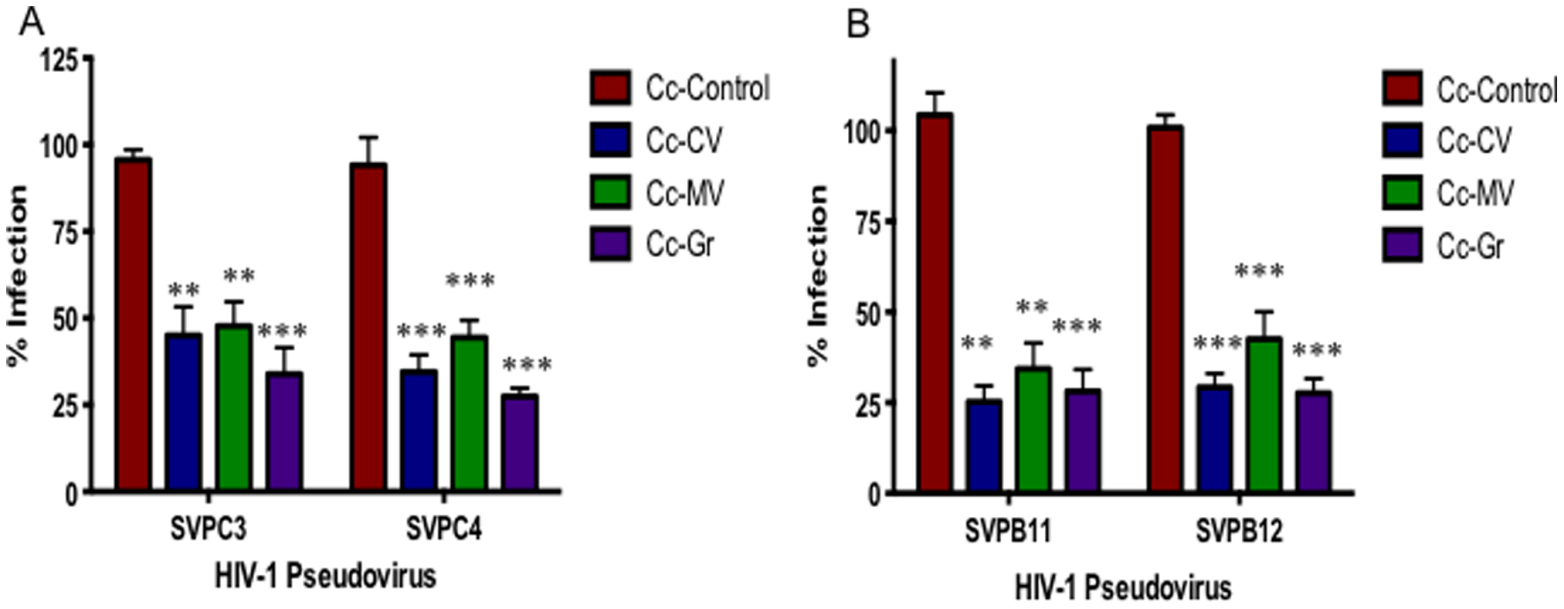
Anti-viral lectin viral blocking assay. Cc-Control, Cc-CV, Cc-MV and Cc-Gr were combined with TZM-bl cells and 200TCID50 clade B (SVPB11, SVPB12) or clade C (SVPC3, SVPC4) HIV-1 pseudovirus and incubated for 48 hours. Infection rate was measured indirectly using a mammalian β-galactosidase assay and is given as a percentage, normalized to wells containing virus+TZM-bl cells, with background for uninfected TZM-bl cells subtracted out. The assay was set up in quadruplicate and repeated a minimum of 3 times. p values represent difference between control and each lectin individually. *p<0.01, **p<0.001.

Cyanovirin-N, microvirin and Griffithsin are carbohydrate binding agents that have been demonstrated to have anti-HIV-1 activity. Cyanovirin-N was isolated from the prokaryote *Nostoc ellipsosporum,* and binds to the mannose repeats on the HIV gp120 protein, potently blocking entry of most HIV clades and subtypes [32]–[34]. Microvirin is also a mannose specific lectin, isolated from *Microcystis aeruginosa,* that has been shown to have anti-HIV activity similar to cyanovirin-N, but has a higher safety profile [35], [36]. Griffithsin was identified and isolated from the red alga *Griffithsia* sp. and has been shown to bind to monosaccharides on the HIV viral envelope, preventing HIV-1 infection [37]–[40]. Each of the anti-HIV lectins were expressed individually on the surface of *C. crescentus* and their ability to prevent HIV-1 infection was measured *in vitro* using HIV-1 pseudovirus. All three anti-HIV lectins provided significant protection from infection with both clade B and clade C viruses (Figure 4). Cc-Gr was the most effective at preventing infection, with protection ranging from 39.8–84.2%. Although Cc-Gr provided the most protection from infection, it was not significantly better than Cc-CV or Cc-MV at preventing HIV-1 infection. It is interesting to note that Cc-CV is more effective at inhibiting infection with clade B viruses compared to clade C viruses, which is likely due to binding affinity to the mannose repeats present on viruses from this clade. Cc-Gr was also tested with pseudoviruses representing clade D and an A/D recombinant virus. Preliminary data indicates that protection from infection is maintained with Cc-Gr across these additional viral clades (data not shown).

### Fusion Inhibitors Provide Significant Protection from HIV-1 Infection *in vitro*

**Figure 5:**
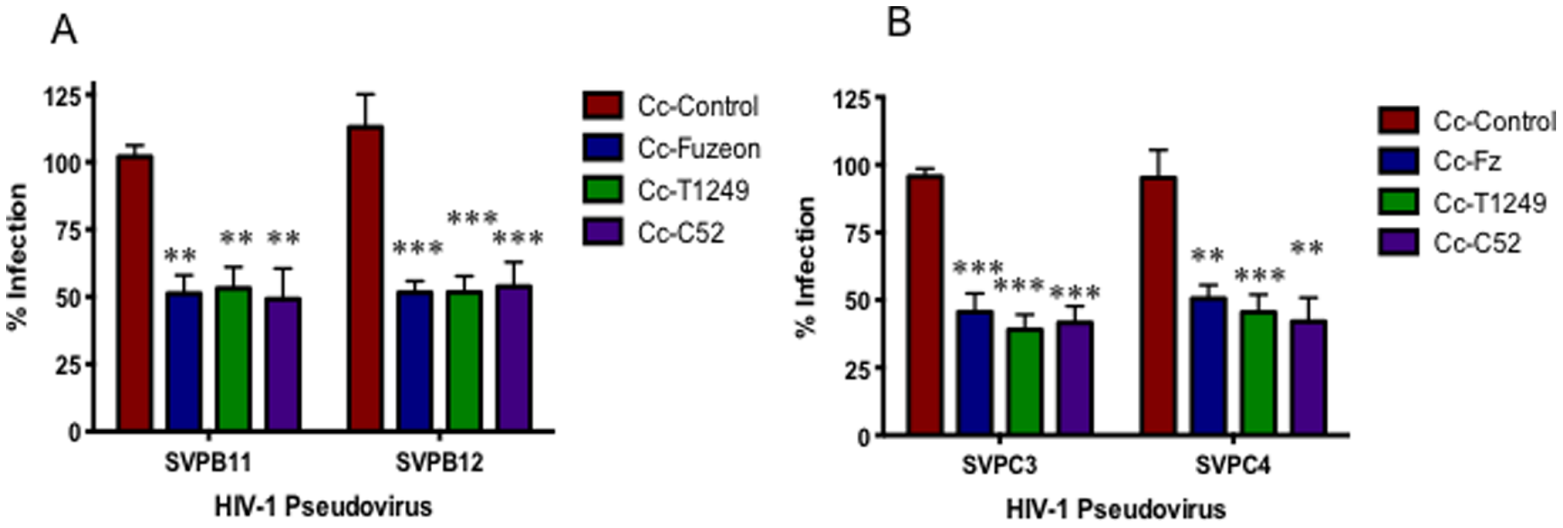
Fusion inhibitor viral blocking assay. Cc-Control, Cc-Fz, Cc-T1249 and Cc-C52 were incubated for 48 hours with TZM-bl cells and 200TCID50 clade B (SVPB11, SVPB12) or clade C (SVPC3, SVPC4) HIV-1 pseudovirus. HIV-1 infection rate was measured indirectly using a mammalian β-galactosidase assay. Infection rate is given as a percentage and normalized to wells containing virus+TZM-bl cells with the background for uninfected TZM-bl cells substracted out. The assay was set-up in quadruplicate and repeated a minimum of 3 times. p values represent the difference between control and each construct individually. *p<0.01, **p<0.001.

Fuzeon (enfuvirtide, T-20) is a 36 amino acid peptide that corresponds to residues 643–678 in the HR2 domain of HIV-1 env protein [41]. Fuzeon competitively binds to HR1 and prevents HR2 from binding, which blocks the formation of the six-helix bundle, thereby preventing fusion of the gp41 protein with the cell surface [42]. T1249 is a second generation variant of Fuzeon, which is more potent and shows a wider activity range against clinical and laboratory isolates [43], [44]. The C52 peptide contains 52 residues of the HIV-1 gp41 glycoprotein, including the region of Fuzeon that has been shown to prevent infection, and C34, a pocket binding region that is active *in vitro* against Fuzeon-resistant viruses [45]. By combining these two regions it is anticipated that greater inhibition of infection will be observed. All three fusion inhibitors were expressed individually on the surface of *C. crescentus* to determine if they could prevent infection with HIV-1, and whether any one provided more protection than the others. All three fusion inhibitors were very effective at preventing HIV-1 pseudovirus infection, with protection ranging from 24–84.8% (Fig. 5). There was no significant difference between each fusion inhibitor in this assay, and protection levels were similar with both viral clades. Cc-T1249 underwent additional testing with viral clade D and an A/D recombinant virus. Protection from infection was maintained at similar levels with these additional viral clades (data not shown).

### Comparing Caulobacter Strains JS4026 and JS4038

The *C. crescentus* protein display system was developed in the Smit lab as a means of expressing useful peptides in high quantities in the S-layer. Previously, some difficulties were encountered in binding of displayed protein to the intended target, especially when affinities were expected to be much less than a typical antibody-antigen interaction. It was our belief that this may be caused by the existence of an extracellular polysaccharide layer (EPS) located exterior to the S-layer [23], which interferes with recombinant protein interactions. To this end, because fucose was known to be present in the EPS but not the LPS (which is required for S-layer attachment) we created knockout mutants of the fucose synthase gene expecting at the least a major modification of the EPS layer. PCR was used to verify that the bacteria was deficient for the fucose synthase gene. Strain JS4038 contains the EPS knockout and produces less or no EPS, as judged by loss of colony mucoidy on sucrose containing solid media. A general trend of an increase in binding efficacy of proteins displayed on the surface of several of our *C. crescentus* constructs has been observed. Specifically, the use of JS4038 strains, including a control (no insert), Cc-MIP1α, Cc-CD4, Cc-CV or Cc-Fz has shown consistently improved viral blocking compared to JS4026 expressing the same peptides. In particular, Cc-MIP1α displayed on JS4038 provided a statistically significant increase in viral blocking (Table S1). Differences between many of the other constructs approached statistical significance. In almost every case, the JS4038 strain construct was shown to function at least as well as, if not better than, the JS4026 equivalent (Table S1). We are therefore confident that the JS4038 strain is a more effective protein display system for these studies.

## Discussion

Herein, we present an improved version of our *C. crescentus* based HIV-1 microbicide. Prior work had described successful protection from infection with HIV-1 pseudovirus representing clade B provided by MIP1α or CD4 encoded bacteria [9]. We extended these experiments by testing HIV-1 pseudovirus representing clade C and observed similar results. In addition, we have generated a variety of novel display constructs, and have shown that they each provide significant protection from HIV-1 infection. The protection level observed with each construct was variable, with the most effective construct, Cc-Gr, providing up to an 84% reduction in HIV-1 infection. Furthermore, our most successful recombinant *C. crescentus* from each inhibitor category underwent additional testing with pseudovirus representing viral clades A, D or an A/D recombinant virus. Cc- MIP1α, Cc-CD4M33F23, Cc-Gr and Cc-T1249 were able to provide similar protection from infection with these additional clades (data not shown). Finally, we compared *C. crescentus* strains JS4026 and JS4038, and demonstrated that the JS4038 strain represents the best viral blocking potential.

Although use of condoms remains an effective means to prevent HIV-1 infection, cultural or societal norms often prevent women from determining condom use [2]. Development of prevention methods that women can control remains an urgent issue. This issue has created interest in the development of microbicides, specifically microbicides that can be topically applied by women in advance of sexual activity and maintain efficacy. Some microbicide formulations have shown promise in a laboratory setting but failed during clinical trials [3]. Recently some success has been observed with the use of tenofovir, a nucleotide reverse transcriptase inhibitor, formulated as a microbicide gel. An initial clinical trial with tenofovir showed promise, providing 39% protection from HIV infection [2], but a second trial was stopped due to inefficacy relative to placebo [46]. The tenofovir trials highlight the potential power of microbicides but also the difficulties in translating laboratory success to clinical use. *In vitro* we have been able to show up to 84% reduction in HIV infection with a single recombinant *C. crescentus*, and it is possible that combining our constructs could provide still higher levels of protection. In addition, it is conceivable that our system combined with antiretroviral based microbicides could further increase efficacy. While we cannot predict the effectiveness of our approach in people, even partial protection will likely make a significant impact. For example, estimates have shown that a microbicide with 60% effectiveness as a single strategy could prevent over 1 million new infections each year [47].

Many microbicides under development for HIV-1 prevention have a high production cost, which could limit their use, particularly in the developing countries where they are most urgently needed. Bacteria-based microbicides have been under investigation for many years. Since *Lactobacilli* are part of the natural flora of the vagina, it was elegantly proposed that engineered *Lactobacillus* would be an excellent choice for this purpose. Various groups have engineered different strains of *Lactobacillus* to express CCR5/RANTES [48], CD4 [6], cyanovirin-N [8], and fusion inhibitors [7], and tested their ability to prevent HIV-1 infection. Efforts to achieve high levels of surface expression or secretion of recombinant proteins in *Lactobacillus* has met with limited success, and the ability to persist as a commensal bacterium in the competitive microbial milieu of the human urogenital system has not been established. In short, additional microbicide strategies seem warranted.

A bacterium based system that can secrete or display blocking proteins at high levels but does not need to compete with other urogenital tract bacteria, because it is expected to be used at high concentrations just before sexual activity (or childbirth), may be more successful. Such a bacterium must be easily and inexpensively produced and can be delivered in a biologically inactive form. The *C. crescentus* display system has these characteristics. A wide variety of proteins have been expressed in the S-layer of *C. crescentus*. The peptides often do not influence expression of the S-layer protein; in this report peptides ranging from 26–121 amino acids were all secreted successfully in this system. Only the mannose binding lectin group of proteins resulted in lower recombinant protein secretion levels. Despite this, they were the group that provided the best protection from HIV infection, indicating that maximum protein expression is not necessarily needed for anti-viral activity in this system. Part of the reason for this lies in the extreme levels of native RsaA secretion, which accounts for as much as 31% of total protein expression in *C. crescentus* [16]. Thus even a significant (e.g., 90%) reduction in secretion levels as the result of a foreign insertion still results in thousands of copies of blocking protein displayed per cell. This high level expression alone suggests that *C. crescentus* deserves serious consideration as an expression platform for microbicide development.

It is anticipated that the final microbicide product generated by these studies would be formulated as a gel or cream that women can apply in advance of sexual activity to protect themselves from HIV infection. The *C. crescentus* protein display system is uniquely able to achieve this goal. *C. crescentus* is a non-pathogenic, freshwater bacterium and is unable to grow at the temperature or salt concentration found in humans, indicating it will neither grow nor compete with the normal vaginal flora. Notably, collaborators have demonstrated that within 7 days post-injection of 2×10^7^ *C. crescentus* intraperitoneally into mice, no bacteria can be isolated from the peritoneal cavity [17]. Thus the approach relies on the high level display of infection inhibiting agents that *C. crescentus* affords, but does not require the prolonged, persistent expression of recombinant proteins needed by approaches involving commensal bacteria. *C. crescentus* are easy and cheap to grow in large quantities, making them feasible for use in developing countries. All of this suggests that *C. crescentus* recombinants are excellent candidates for development into microbicides.

Clade B and C pseudoviruses were primarily used for these studies because these clades account for approximately 62% of worldwide HIV-1 infections, including sub-Saharan Africa (clade C) and Southeast Asia (clade B) as well as Western Europe and North and South America. The efficacy of the *C. crescentus* display system was maintained between clades B and C, so it can be expected that this system will provide protection from additional HIV-1 clades with relatively similar efficacy. Preliminary studies with clade A and D pseudoviruses, and an A/D recombinant virus support the maintenance of efficacy among viral clades. As the majority of our display constructs target well conserved features of the HIV-1 attachment and entry process, we anticipate that these display constructs would maintain protection with the all of HIV-1 clades. Although the HIV-1 pseudoviruses are only capable of a single-cycle infection they use CD4 and CCR5 to enter cells identically to HIV and are an appropriate and accepted method for viral entry studies. Initial studies from the Cavacini lab indicate that the *C. crescentus* recombinant display system is able to prevents infection of PBMCs from live HIV-1 (unpublished data).

The creation of many new *C. crescentus* display constructs was described in this paper. Creating constructs displaying anti-HIV lectins and fusion inhibitors was an important step for a number of reasons. First, it provides further evidence that *C. crescentus* appears to be easily modified to express a variety of structurally different anti-HIV peptides. Further, a large reduction in HIV-1 infection occurred using individually displayed peptides. When *C. crescentus* expressing CD4 or MIP1α were combined, a significant increase in protection from infection occurs [9]. This finding leads us to anticipate that combining multiple constructs, either on a single *C. crescentus* or several strains mixed together, might further reduce infection rates.

Since *C. crescentus* is a gram-negative bacterium there is concern that it could initiate an immune response when applied topically. However, immune activation may not be an issue because *C. crescentus* appears relatively non-immunogenic. *C. crescentus* has been injected into immune competent mice and no adverse events were observed [17]. In addition, within as little as ten days post-injection, no *C. crescentus* could be detected within the peritoneum [17] The lipid A portion of the lipopolysaccharide of *C. crescentus* is 1000-fold less immunogenic than that of LPS normally found on *E. coli* [18]. Although we have not topically applied *C. crescentus* to the vaginal tract, adverse events are not anticipated, and the mucosal immune response to topical *C. crescentus* application is currently under investigation. In addition, the final microbicide product would contain inactivated *C. crescentus*, which should further increase the safety profile.

In summary, the *C. crescentus* display system represents an exciting new approach in the HIV-1 microbicide field with large potential. We have generated a sufficient number of constructs to now address the clinical relevance and safety of this approach in animal studies, such as an HIV susceptible humanized mouse model.

## Supporting Information

Table S1 JS4026 and JS4038 strains were compared side-by-side in the described viral blocking assay. Results represent mean with the range provided. Experiments were set up in quadruplicate and performed a minimum of 3 times.
(PDF)
