# Supplementary material for: Development of an HIV-1 Microbicide Based on Caulobacter crescentus: Blocking Infection by High-Density Display of Virus Entry Inhibitors
Source: PLoS One. 2013 Jun 19;8(6):e65965. doi: 10.1371/journal.pone.0065965 (PMC3686833; doi:10.1371/journal.pone.0065965)
Supplement: Table S1 — JS4026 and JS4038 strains were compared side-by-side in the described viral blocking assay. Results represent mean with the range provided. Experiments were set up in quadruplicate and performed a minimum of 3 times. (PDF) [file pone.0065965.s001.pdf]

Construct	Cc-Control		Cc-MIP1α		Cc-CD4		Cc-CV		Cc-Fz	
Strain										
SVPB11	121.9 (98.1-148.2)	114.9 (93.5-138.7)	50.3 (35.6-60)	43.1 (27.5-69)	70.4 (59.7-79.3)	67.7 (62.4-71.5)	36.3 (28.1-51.7)	28.5 (18.9-37.2)	45.8 (22.4-60.4)	52.7 (34-78.6)
SVPB12	118.2 (92.4-131)	111 (92.4-131)	44.7 (30.3-67.5)	48.2 (42.2-54.5)	67.2 (52.4-86.5)	71.5 (63.5-78)	34.7 (18-61.8)	23.5 (14-35.1)	50 (34.3-81.8)	47.8 (34.5-58.7)
SVPC3	116.2 (90.9-162)	72.3 (46.2-87.2)	53.2 (41.6-71.8)	32.4 (15.9-44.8)	72.3 (46.2-87.2)	74.2 (54.5-88)	40.9 (27.3-45.7)	29.6 (19.1-40)	59.9 (30.5-87.1)	43.2 (19.6-61.1)
SVPC4	122.7 (91.9-164.6)	118.5 (97.4-136.6)	56.7 (44.5-66.8)	33.9 (31.9-37.1)	70.7 (61.2-79.1)	64.3 (61-67.5)	44.5 (27.4-56.1)	25.8 (21-30.8)	59.5 (49.1-76.8)	56.6 (36.9-70.3)

Legend: JS4026 and JS4038 strains were compared side-by-side in the described viral blocking assay. Results represent mean with the range provided. Experiments were set up in quadruplicate and performed a minimum of 3 times
